# Investigation of periodontitis, halitosis, xerostomia, and serological characteristics of patients with osteoarthritis and rheumatoid arthritis and identification of new biomarkers

**DOI:** 10.1038/s41598-024-55004-w

**Published:** 2024-02-21

**Authors:** Yeon-Hee Lee, Seung-Jae Hong, Gi-Ja Lee, Seung-Il Shin, Ji-Youn Hong, Sang Wan Chung, Yeon-Ah Lee

**Affiliations:** 1 Department of Orofacial Pain and Oral Medicine, Kyung Hee University Dental Hospital, #613 Hoegi-dong, Dongdaemun-gu, Seoul, 02447 Korea; 2 Division of Rheumatology, Department of Internal Medicine, School of Medicine, Kyung Hee University, Dongdaemun-gu, Seoul, 02447 Korea; 3 Department of Biomedical Engineering, Kyung Hee University, Dongdaemun-gu, Seoul, 02447 Korea; 4 Department of Periodontology, Periodontal-Implant Clinical Research Institute, School of Dentistry, Kyung Hee University, Dongdaemun-gu, Seoul, 02447 Korea

**Keywords:** Rheumatoid arthritis, Osteoarthritis, Halitosis, Xerostomia, Anti-CCP antibody, Biomarker, Biomarkers, Health care, Medical research, Rheumatology, Signs and symptoms

## Abstract

Rheumatoid arthritis (RA) and osteoarthritis (OA) are two different types of arthritis. Within RA, the subsets between seronegative RA (snRA) and seropositive RA (spRA) represent distinct disease entities; however, identifying clear distinguishing markers between them remains a challenge. This study investigated and compared the oral health conditions in patients with RA and OA to clarify the differences from healthy controls. In addition, we investigated the serological characteristics of the patients, the factors that distinguished patients with RA from those with OA, and the main factors that differentiated between snRA and spRA patients. A total of 161 participants (mean age: 52.52 ± 14.57 years, 32 males and 129 females) were enrolled in this study and categorized as: normal (n = 33), OA (n = 31), and RA (n = 97). Patients with RA were divided into the following two subtypes: snRA (n = 18) and spRA (n = 79). Demographics, oral health, and serological characteristics of these patients were compared. The prevalence of periodontal diseases was significantly higher in patients with OA (100%) and RA (92.8%) than in healthy controls (0.0%). However, the presence of periodontal diseases was not utilized as a distinguishing factor between OA and RA. Xerostomia occurred more frequently in patients with RA (84.5%) than in patients with OA (3.2%) and healthy controls (0.0%) (all *p < *0.001). ROC analysis revealed that periodontal disease was a very strong predictor in the diagnosis of OA compared to healthy controls, with an AUC value of 1.00 (*p < *0.001). Additionally, halitosis (AUC = 0.746, 95% CI 0.621–0.871, *p < *0.001) and female sex (AUC = 0.663, 95% CI 0.529–0.797, *p < *0.05) were also significant predictors of OA. The strongest predictors of RA diagnosis compared to healthy controls were periodontal diseases (AUC = 0.964), followed by xerostomia (AUC = 0.923), age (AUC = 0.923), female sex (AUC = 0.660), and halitosis (AUC = 0.615) (all *p < *0.05). Significant serological predictors of RA were anti-CCP Ab (AUC = 0.808), and RF (AUC = 0.746) (all *p < *0.05). In multiple logistic regression analysis, xerostomia (odds ratio, OR: 8124.88, 95% CI 10.37–6368261.97, *p*-value = 0.008) and Anti-CCP Ab (OR: 671.33, 95% CI 2.18–207,074.02, *p* = 0.026) were significant predictors for RA compared to OA. When diagnosing spRA compared to snRA, anti-CCP Ab (AUC = 1.000, *p < *0.001) and RF (AUC = 0.910, 95%CI 0.854–0.967, *p < *0.001) had outstanding predictive performances. Therefore, clinicians and researchers should thoroughly evaluate the oral status of both OA and RA patients, alongside serological factors, and consider these elements as potential predictors.

## Introduction

Arthritis is a serious and common chronic disease that affects 23% of the world’s population^1^. The documentation and medical explanations of inflammatory arthritis date back to the writings of Hippocrates^2^. Arthritis affects adults of all ages, and its prevalence reportedly increases with age. Arthritis has been reported to occur in 7.1% adults aged 18–44 years and approximately one-third (30.5%) of adults aged 45–64 years^3^. A total of 34% adults belonging to > 65 years age group have been reportedly diagnosed with arthritis^4^. Reduced physical activity occurs in 44% of patients with arthritis. The disease reduces the quality of life of an individual and imposes significant economic burden on the society^5^. Arthritis is an umbrella term that refers to inflammation of the joints and has various subcategories. Therefore, appropriate diagnosis, treatment, and management are required.

The term “arthritis” refers to more than 100 conditions that involve gradual erosion of and pain in the joints. The most common forms of arthritis are osteoarthritis (OA) and rheumatoid arthritis (RA)^6^. Approximately 80–90% of adults aged > 65 years are likely to have OA, even if there are asymptomatic^7^. OA and RA are joint diseases with different etiologies. OA is characterized by progressive destruction of cartilage and erosion of bone, which leads to pain, swelling, and stiffness. On the other hand, RA is an autoimmune disease in which the body’s immune system attacks the joint tissues. Risk factors for OA include weight, age, genetics, and history of joint injury^8^. More specifically, factors such as old age, female sex, hormone profiles specific to sex, chronic joint overloading, and obesity contribute to the pathogenesis of OA^9–11^. Females are approximately twice as likely to develop degenerative OA in all joints compared to males, especially the knees^12^.

RA is the most common systemic autoimmune disease affecting about 0.5–1.0% of the world’s population^13,14^. In this disease, the immune system attacks the joints, resulting in joint inflammation and thickening of the tissue surrounding the joints. This causes swelling and pain inside and outside the joints, thereby resulting in permanent disability^15^. Patients with RA face a high financial burden because of their signs and symptoms, especially due to unemployment, disability, high medical costs, and management of their condition^16^. Although the exact pathogenesis of RA is unknown, it is widely believed that both genetic and environmental factors play important roles in the pathogenesis of this disease^17^. The primary risk factors for RA include female sex, age, and family history^6^. The female-to-male ratio of RA prevalence has been reported to be approximately 3:1^18^. RA usually develops between the ages of 40–60 years^6^. Research is warranted to delineate the differences between RA and OA; however, investigations and comparisons of oral health and disease in these patients have been limited.

RA is classified into seronegative (snRA) and seropositive (spRA) subsets on the basis of presence or absence of seropositivity for rheumatoid factor (RF) and anti-citrullinated protein antibodies (anti-CCP Ab)^19^. snRA: spRA reportedly occurs at a ratio of 1:3^20^. Anti-CCP Ab is a hallmark of RA and, together with RF, it forms a part of the 2010 American College of Rheumatology (ACR)/European League Against Rheumatism (EULAR) classification criteria^21^. In general, patients with snRA require to demonstrate more clinical symptoms than those with spRA to be classified as having RA according to the 2010 ACR/EULAR criteria^22^. As a result, snRA is more likely to be diagnosed at a later and more advanced stage than spRA. All patients benefit from early diagnosis and treatment. Early diagnosis is important in patients with snRA as it is in those with spRA^23^. The biggest obstacle to early diagnosis is the lack of biomarkers that distinguish snRA from spRA or predict each subset.

OA and RA share similar clinical characteristics; however, the mechanisms of occurrence and treatment protocols for the two entities are different. Moreover, the prognoses according to treatment also varies. The two subsets of RA, snRA and spRA, are different disease entities; however, there is a lack of clear distinguishing markers. Nevertheless, since early detection and diagnosis are important for effective control of diseases, research and development of novel biomarkers are crucial. In this study, we hypothesized that the decrease in salivary flow rate will be more significant in patients with RA than in those with OA, and that the resulting halitosis and occurrence of periodontal diseases would be major oral indicators that distinguish patients with RA from those with OA. We also hypothesized that there exists an oral health-related and/or serological biomarker that differentiates snRA from spRA. To test this hypothesis, we investigated periodontal diseases (gingivitis and periodontitis) and halitosis using VSC measurement, unstimulated whole saliva flow rate, and xerostomia in patients with OA and RA and compared it with the values obtained for healthy controls to sought a novel biomarker. did. In addition, hematological characteristics, including anti-CCP Ab, RF, CRP, and ESR, for traditional distinction between patients with OA and RA and between patients with snRA and spRA were also investigated. Therefore, we aimed to investigate the oral health of these patients, elicit their oral characteristics, and identify new oral biomarkers for OA and RA.

## Methods

### Study population

For this case–control study, patients aged ≥ 18 years with RA (n = 97) or OA (; n = 31) were enrolled prospectively at the Rheumatology Clinic of Kyung Hee University Hospital between December 2021 and October 2022. Diagnosis of RA was made in accordance with the 1987 ACR classification criteria or 2010 ACR/EULAR classification criteria for RA^24^. snRA (n = 18) was defined as the absence of both RF and anti-CCP Abs, whereas spRA (n = 79) was defined as the presence of at least one of the two antibodies. OA was defined as pain in either or both knees and/or hands on majority of the days of the previous three months in combination with radiological changes according to the ACR criteria^25^. Two experienced rheumatologists (YAL and SJH) made the diagnoses of OA and RA. Periodontal assessments were conducted by two experienced periodontists (JYH and SIS), and radiographic examinations were performed to confirm the diagnosis for each participant. Calibration exercises by measuring clinical attachment loss (CAL) in 10 patients at 24-h intervals were conducted by two trained examiners. Intra-class correlation coefficients (ICCs) of 0.80 and 0.89 were estimated in intra-examiner reproducibility measurements. Inter-examiner ICCs for CAL were 0.80 and 0.81 in each measurement. When there was a disagreement, a unified consensus was reached through discussions. The measurement of parameters related to periodontal disease and diagnosis, including gingivitis and periodontitis, were in accordance to our previously described methods^26^.

For sample size calculation, G*Power software (ver. 3.1.9.7; Heinrich-Heine-Universität Düsseldorf, Düsseldorf, Germany) was used. It was found that 100 participants (α level = 0.05, power = 0.90, and effect size = 0.5) with an actual target of at least 30 per group were suitable for statistical analysis, and a total of 161 participants were recruited.

### Study design

Patients with OA and RA who visited the Rheumatology Clinic of Kyung Hee University Hospital, met the aforementioned diagnostic conditions, and voluntarily expressed their intention to participate in this study formed our study population. Clinical data collection, physical examinations, and blood tests were performed for all patients with OA and RA by two rheumatologists. The patient then visited Kyung Hee University Dental Hospital and underwent oral examination, salivary flow rate test, and VSC measurements for diagnosis of halitosis. During this process, approximately 20% of the participants dropped out due to reasons such as long and complicated research protocol, pain and discomfort during periodontal examination, and reluctance to spit out saliva. Moreover, it was not feasible to make a diagnosis of periodontal disease in patients with less than 20 remaining teeth. Therefore, a total of 128 patients with OA or RA were finally included in this study.

Healthy controls included individuals who did not have any major systemic diseases or were not regularly taking medications for physical or psychological conditions^27^. Oral examinations, salivary flow rate tests, and VSC measurements were performed in 31 healthy controls; however, blood tests were excluded.

### Ethical consideration

All participants were given adequate information about the study. The study protocols were approved by the Ethics Committee of Kyung Hee Clinical Research Institute, Kyung Hee University Medical Center (IRB on recruitment of normal controls: IRB no. KH-DT20030; IRB on recruitment of RA and OA patients: KHUH-2021–08-074) and conducted in accordance with the tenets of Declaration of Helsinki. Informed consent was obtained from all the participants prior to the commencement of the study.

### Serological tests

Mean values of hematological indicators, including anti-CCP Ab, RF, CRP, and ESR, were measured in the serum of all patients with OA and RA. The values were dichotomized (positive when above the threshold, negative when below the threshold) for AUC analyses. Anti-CCP Ab ≥ 20 IU/mL^28^, RF ≥ 20 IU/mL^29^, CRP > 0.5 mg/dL^30^, ESR > 15 mm/h in males, and ESR > 20 mm/h in females^31^ were considered abnormal or elevated.

### Diagnosis of gingivitis and periodontitis

The case definitions for healthy periodontium, gingivitis, and chronic periodontitis were based on criteria established in the 2017 World Workshop on the Classification of Periodontal Diseases^32^. A clinically healthy periodontium was diagnosed when subjects exhibited probing depths (PD) ≤ 3 mm, bleeding on probing (BOP) at sites < 10%, and no clinical attachment loss (CAL). Healthy controls were defined as individuals without major systemic diseases or regular medication intake for physical/psychological issues. Participants displaying BOP at ≥ 10% of sites and PD ≤ 3 mm across all sites were categorized as gingivitis. The periodontitis was diagnosed with the following criteria: (1) interdental CAL > 5 mm at the site of greatest loss; (2) radiographic bone loss exceeding the mid 1/3 of the root; (3) tooth loss due to periodontal disease; and (4) a maximum PD of ≥ 6 mm affecting ≥ 30% of teeth, corresponding to stages III and IV in the generalized pattern.

### Salivary flow rate and xerostomia

Prior to saliva collection, the participants were instructed to refrain from caffeine and/or nicotine for at least 4 h and alcohol for at least 24 h. Saliva was collected between 9:00 and 11:00 a.m. to minimize circadian differences. Unstimulated whole-saliva flow rate was determined by measuring the amount of saliva collected using spitting method for 10 min while the patient was resting^27^. In normal individuals, the unstimulated salivary flow rate was measured for 10 min according to a previously described method. However, patients with OA and RA complained that they had difficulty producing and expelling saliva; therefore, the spitting time was reduced from 10 to 5 min when measuring the unstimulated salivary flow rate. Salivary flow rate was expressed in mL/min^33^. As for the existence of self-reported xerostomia, participants answered yes/no to the question, “Over the past month, have you ever had dry mouth or discomfort in daily activities such as eating or swallowing saliva due to dry mouth?”.

### VSC measurement

Hydrogen sulfide (H_2_S) and methyl mercaptan (CH_3_SH) levels in mouth air of the participants were measured using a portable gas chromatograph (TwinBreasor II, IsenLab, Gyeonggido, Korea) equipped with a flame photometric detector. Briefly, 10 mL sample of the participant’s mouth air was passed through an electrolytic sensor, the concentrations of H_2_S and CH_3_SH were detected, indicating a peak level in parts per billion (ppb) on the digital scale of the monitor. Halitosis measurements were performed between 9:00 and 11:00 a.m. in a well-ventilated laboratory environment with no olfactory, visual, or auditory stimuli that could interfere with accurate measurements. On the day of measurement, the participants were asked to maintain their usual eating habits and limit the use of alcohol, cosmetics, and perfumes that could affect the VSC levels. The concentrations of H_2_S and CH_3_SH and their sum (VSC sum) were expressed in ppb^27^. Presence of halitosis was determined according to the findings of a previous study on Korean participants that sought the cut-off value for halitosis diagnosis (65.79 ppb for women and 79.94 ppb for men)^34^.

### Statistical analysis

Data were analyzed using the Statistical Package for Social Sciences (SPSS) for Windows (version 26.0; IBM Corp. Armonk, NY, USA). Descriptive statistics were reported as mean ± standard deviation or numbers with percentages, as appropriate. To analyze the distribution of discontinuous data, we used the χ^2^ and Bonferroni tests for equality of proportions. Analysis of variance and Tukey’s post-hoc tests were used to compare the values of the parameters among the healthy controls, OA, and RA groups. The t-test was used to compare the parameter values between the OA and RA groups and between the snRA and spRA subgroups. Multiple logistic regression analysis was utilized to examine factors contributing to the prediction of the presence of RA compared to OA. In patients with OA and RA, correlations between oral diseases, including halitosis, periodontal disease, and xerostomia, and positive results for serological indicators (anti-CCP Ab, RF, CRP, and ESR) were obtained using Cramer’s V analysis. Cramer's V is a measure of association between two categorical variables that returns a value between 0 (weak) and 1 (strong). To demonstrate the performance at the classification threshold (above the mean value of each laboratory parameter), a receiver operating characteristic (ROC) curve was plotted, and the area under the ROC curve (AUC) value was calculated for each model. As the rule of thumb for interpreting the AUC values, the following criteria were used: AUC = 0.5 (no discrimination), 0.7 < AUC ≤ 0.8 (acceptable), 0.8 < AUC ≤ 0.9 (excellent), and AUC > 0.9 (outstanding discrimination)^35^. Statistical significance was set at a two-tailed p-value < 0.05 for all analyses.

## Results

### Demographics

Significant difference was observed among the groups in terms of age. The average age of patients with OA (63.52 ± 7.58 years) and RA (56.98 ± 12.30 years) was significantly higher than that of healthy controls (38.82 ± 14.23 years). Moreover, it was also observed that patients with OA were significantly older than those with RA. The age of the patients was significantly higher (all *p < *0.05). In terms of sex distribution, the ratio of female patients with OA (87.1%) and RA (86.6%) was significantly higher than that of healthy controls (54.5%) (*p < *0.001) (Table 1).

**Table 1:** Comparison of demographics and oral health of patients with OA and RA.

	Healthy controls (n = 33)	OA (n = 31)	RA (n = 97)	*p*-value	Post-hoc
	Mean ± SD or n (%)	Mean ± SD or n (%)	Mean ± SD or n (%)		
Demographics					
Age (years)^a^	38.82 ± 14.23	63.52 ± 7.58	56.98 ± 12.30	< 0.001***	OA > RA, OA > Control, RA > Control
Sex^b^					
Male	15 (45.5%)	4 (12.9%)	13 (13.4%)	< 0.001***	Female: OA > Control, RA > Control
Female	18 (54.5%)	27 (87.1%)	84 (86.6%)		
Smoking habit^b^	1 (3.0%)	3 (9.7%)	12 (12.4%)	0.301	
Non-periodontal disease^b^	33 (100.0%)	0 (0.0%)	7 (7.2%)	< 0.001***	
Periodontal diseases	0 (0.0%)	31 (100%)	90 (92.8%)		OA > Control, RA > Control
(1) Gingivitis^b^	0 (0.0%)	4 (12.9%)	19 (19.6%)	< 0.001***	OA > Control, RA > Control
(2) Periodontitis^b^	0 (0.0%)	27 (87.1%)	71 (73.2%)	< 0.001***	OA > Control, RA > Control
VSC level (ppb)					
(1) H_2_S^a^	14.97 ± 31.22	83.13 ± 81.14	45.98 ± 66.86	< 0.001***	OA > RA, OA > Control, RA > Control
(2) CH_3_SH^a^	5.72 ± 14.10	36.77 ± 31.32	23.34 ± 37.82	0.001**	OA > Control, RA > Control
(3) VSC sum^a^	19.80 ± 40.19	116.32 ± 107.50	70.51 ± 95.53	< 0.001***	OA > RA, OA > Control, RA > Control
Halitosis^b^	4 (12.1%)	19 (61.3%)	34 (35.1%)	< 0.001***	OA > RA, OA > Control, RA > Control
Salivary flow rate(mL/min)^a^	1.12 ± 0.33	0.30 ± 0.74	0.16 ± 0.32	< 0.001***	OA > RA, Control > OA, Control > RA
Xerostomia^b^	0 (0.0%)	1 (3.2%)	82 (84.5%)	< 0.001***	RA > Control, RA > OA

^a^ Results were obtained using analysis of variance and post hoc analysis. ^b^ Results were obtained using two-sided Chi-square analysis. SD, standard deviation; VSC, volatile sulfur compound; Control, healthy control; OA, osteoarthritis; RA, rheumatoid arthritis; VSC, volatile sulfur compound. Statistical significance was set at p-value < 0.05. *p < 0.05, **p < 0.01, ***p < 0.001, significant values are mentioned in bold.

### Periodontal diseases, halitosis, and xerostomia in patients with OA and RA

The incidence of periodontal diseases, halitosis, and xerostomia was investigated in all healthy controls and patients with OA and RA. There was a significant difference in the distribution of periodontal diseases among the groups. The prevalence of gingivitis and periodontitis in healthy controls was 0.0%. The prevalence of periodontal diseases was significantly higher in patients with OA (100%) and RA (92.8%) than in healthy controls (*p < *0.001). When analyzing the distribution of periodontal diseases by categorizing them into gingivitis and periodontitis, gingivitis was distributed more frequently in patients with OA (12.9%) and RA (19.6%) than in healthy controls (0.0%) (*p < *0.001). Additionally, periodontitis was also found at a significantly higher rate in patients with OA (87.1%) and RA (73.2%) than in healthy controls (0.0%) (*p < *0.001). However, the difference in distribution of both gingivitis and periodontitis between patients with OA and RA was not significant. Most patients with OA and RA (100% and 92.8%, respectively) had periodontal diseases. Specifically, periodontitis occurred at a significantly higher rate than gingivitis in patients with OA (87.1% vs. 12.9%) or RA (73.2% vs. 19.6%). Periodontal diseases occurred at a higher rate in both patients with OA and RA; therefore, periodontal diseases were not considered as indicators for distinguishing between patients with OA and RA. The distribution of the smoking habit, determined by binary responses (yes or no) from patients, showed no significant difference between groups: healthy control (3.0%), OA (9.7%), and RA (12.4%) (*p* = 0.301) (Table 1).

**Figure 1:**
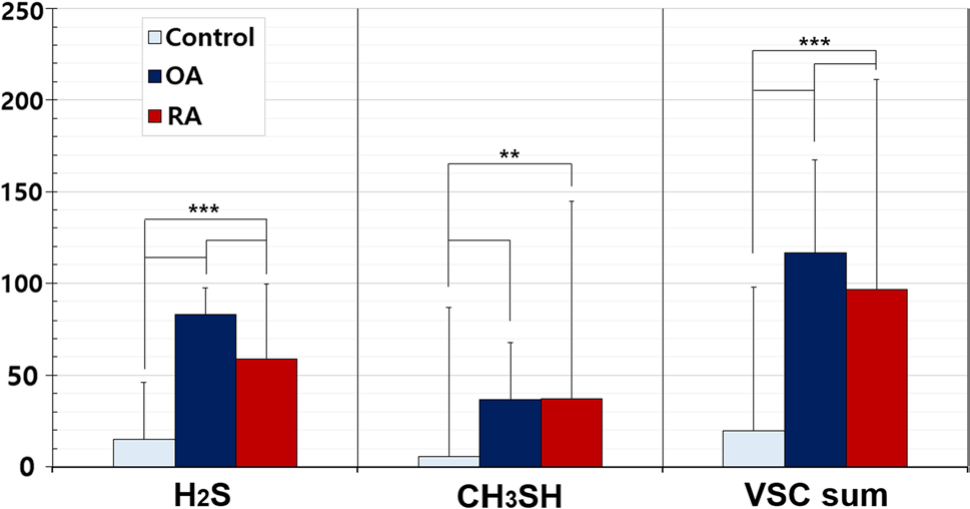
Comparison of VSC levels among healthy controls, patients with OA, and patients with RA. Control: heathy controls, OA: osteoarthritis, RA: rheumatoid arthritis. Statistical significance was set at p-value < 0.05. **p < 0.01, ***p < 0.001. Significant values are marked in bold.

We investigated the VSC levels and halitosis distribution in patients with OA and RA, and compared it with the healthy controls. The VSC levels, H_2_S, CH_3_SH, and their sum (VSC sum) were all significantly higher in patients with OA and RA than in healthy controls (Fig. 1). Moreover, H_2_S and VSC sum values were significantly higher in patients with OA than in those with RA. The incidence of halitosis was significantly higher in patients with OA (61.3%) and RA (35.1%) than in healthy controls (12.1%), and the order was OA > RA > healthy controls (*p < *0.001). Therefore, halitosis and VSC levels can be used as indicators to distinguish patients with OA from healthy controls or those with RA.

Salivary flow rate significantly was found to be decreased in the following order: healthy controls (1.12 ± 0.33 mL/min) > patients with OA (0.30 ± 0.74 mL/min) > patients with RA (0.16 ± 0.32 mL/min) (*p < *0.001). Xerostomia occurred more frequently in patients with RA (84.5%) in comparison to those with OA (3.2%) and healthy controls (0.0%) (*p < *0.001). The order of occurrence of xerostomia was RA > OA > healthy controls (Table 1).

**Figure 2:**
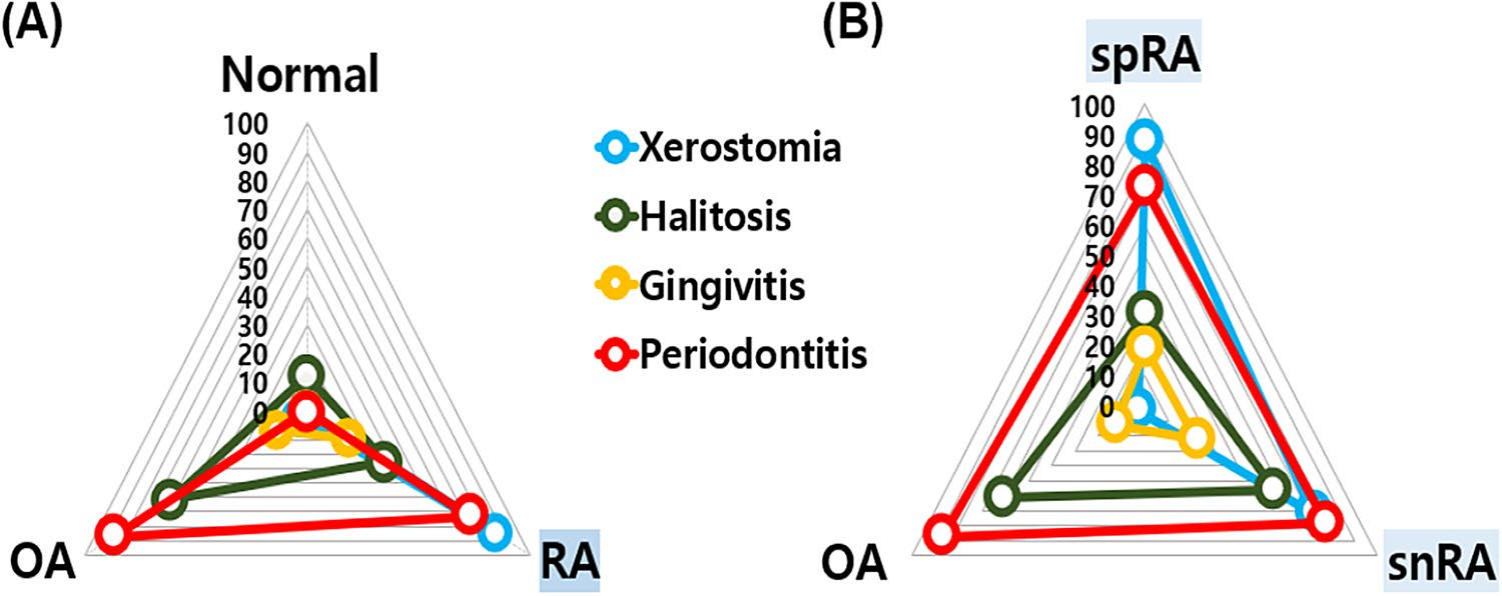
Distribution of oral diseases in patients with OA and RA. (**A**) Gingivitis and periodontitis were significantly higher in the OA and RA patient groups than in the normal controls; halitosis was significantly higher in the OA patient group, and xerostomia was significantly higher in the RA patient group than in the other groups. (**B**) Halitosis was significantly higher in patients with snRA than in those with spRA. OA: osteoarthritis, RA: rheumatoid arthritis, snRA: seronegative RA, spRA: seropositive RA.

Gingivitis and periodontitis were significantly more common in the OA and RA patient groups than in normal controls, halitosis was significantly more common in the OA patient group, and xerostomia was significantly more common in the RA patient group than in other groups (Fig. 2A).

### OA and RA predicted by H_2_S, CH_3_SH, and VSC sum

**Figure 3:**
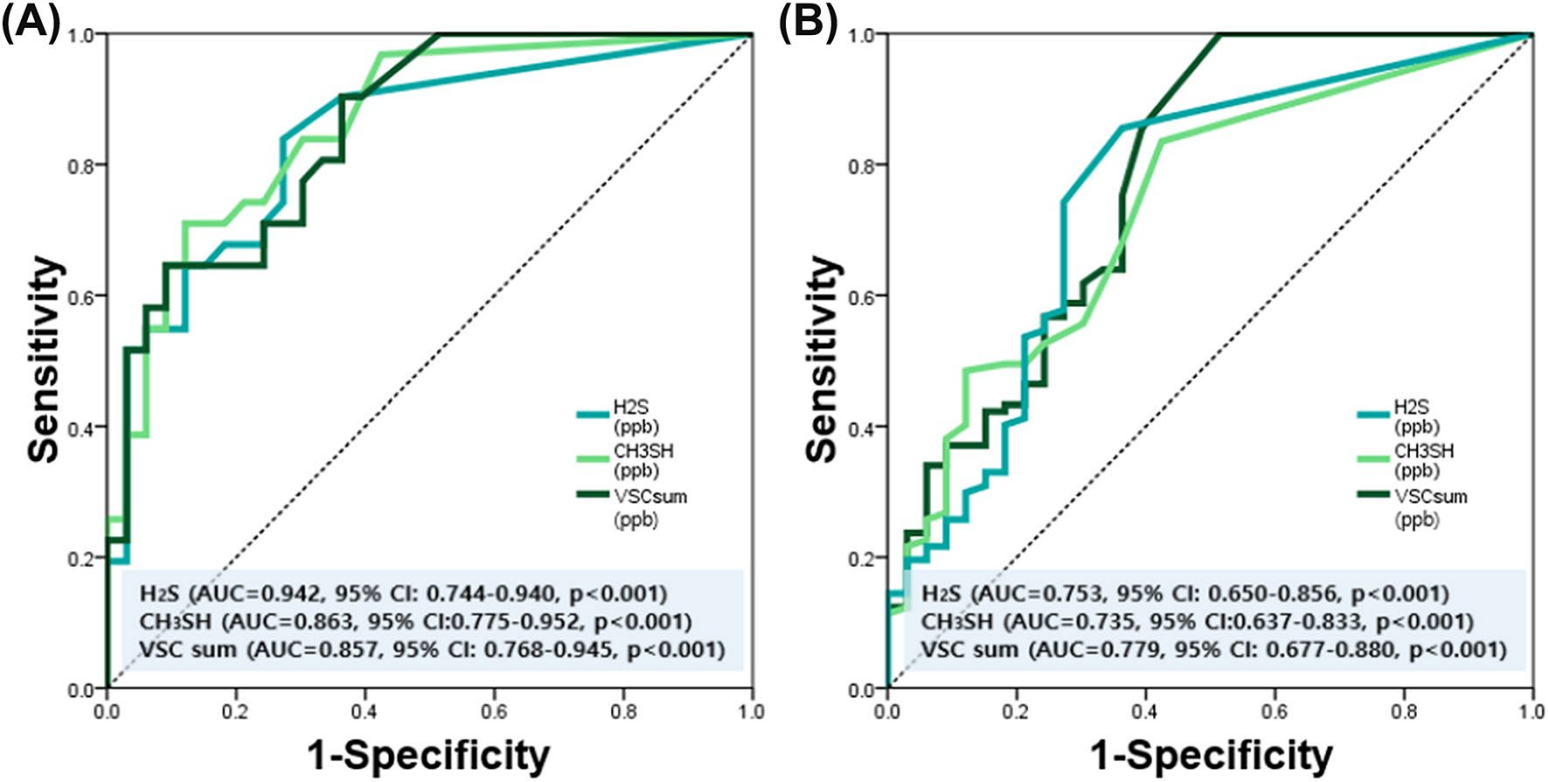
Predicting OA and RA using H_2_S, CH_3_SH, and VSC level. (**A**) Predicting OA and (**B**) RA.

AUC curve was used to check the predictive power when using H_2_S, CH_3_SH, and VSC sum to predict OA and RA (Fig. 3). When predicting OA using H_2_S, the prediction accuracy was outstanding at AUC = 0.942 (95% CI 0.744–0.940, *p < *0.001), which was 94.2%. When predicting OA using CH_3_SH, the AUC was 0.863 (95% CI 0.775–0.952, *p < *0.001), and the prediction accuracy was excellent at 86.3%. When predicting OA using the VSC sum, the AUC was 0.857 (95% CI 0.768–0.945, *p < *0.001), which also showed excellent performance.

When predicting RA using H_2_S, the AUC was 0.753 (95% CI 0.650–0.856, *p < *0.001). When predicting RA using CH_3_SH, the AUC was 0.735 (95% CI 0.637–0.833, *p < *0.001); and when predicting RA using the VSC sum, the AUC was 0.779 (95% CI 0.677–0.880, *p < *0.001). When RA was predicted using H_2_S, CH_3_SH, and VSC sum, 0.7 < AUC < 0.8 was found to be an acceptable performance. In summary, the prediction accuracy of OA was better than that of RA when using H_2_S, CH_3_SH, and VSC sum levels.

### Hematological characteristics of patients with OA and RA

The mean values of anti-CCP Ab, RF, and CRP were higher in patients with OA than in those with RA (all *p < *0.05). Moreover, there was no significant difference in the ESR of patients with OA and RA. Anti-CCP Ab was significantly higher in patients with RA than in those with OA (79.96 ± 331.43 IU/mL vs. 434.52 ± 608.54 IU/mL, *p < *0.001). RF was significantly higher in patients with RA compared to patients with OA (42.15 ± 85.88 IU/mL vs. 111.41 ± 181.82 IU/mL, *p < *0.01). ESR of patients with OA (19.65 ± 13.11 mm/h) was lower than that of those with RA (23.29 ± 15.69 mm/h); however, the difference was not statistically significant.

In cases of abnormal increases exceeding the threshold (positive), anti-CCP Ab was significantly higher in patients with RA than in those with OA (21.7% vs. 80.4%, *p < *0.001). Similarly, RF was significantly higher in patients with RA than in those with OA (15.4% vs 67.0%, *p < *0.001). There was no significant difference in terms of CRP (3.2% vs. 8.2%, *p* = 0.687) and ESR (45.2% vs. 56.7%, *p* = 0.304) between the two groups (Table 2).

**Table 2:** Comparison of serological characteristics, BMI, and diabetes mellitus between OA and RA patients.

	OA (n = 31)	RA (n = 97)	*p*-value
	Mean ± SD or n (%)	Mean ± SD or n (%)	
Anti-CCP Ab (IU/mL)^a^	79.96 ± 331.43	434.52 ± 608.54	< 0.001***
Anti-CCP Ab positive^b^	5 (21.7%)	78 (80.4%)	< 0.001***
RF(IU/mL)^a^	42.15 ± 85.88	111.41 ± 181.82	0.007**
RF positive^b^	4 (15.4%)	65 (67.0%)	< 0.001***
CRP (mg/dL)^a^	0.22 ± 0.42	0.83 ± 0.37	< 0.001***
CRP positive^b^	1 (3.2%)	8 (8.2%)	0.687
ESR (mm/hr)^a^	19.65 ± 13.11	23.29 ± 15.69	0.205
ESR positive^b^	14 (45.2%)	55 (56.7%)	0.304
BMI (kg/m^2^)^a^	24.58 ± 3.33	23.81 ± 3.84	0.321
Overweight (≥ 25 kg/m^2^)^b^	25 (80.6%)	60 (61.9%)	0.041*
Obesity (≥ 30 kg/m^2^)^b^	1 (3.2%)	4 (4.1%)	0.137
Diabetes mellitus^b^	9 (29.0%)	10 (10.3%)	0.015*

^a^ Results were obtained using t-test. ^b^ Results were obtained using two-sided Chi-square analysis. OA, osteoarthritis; RA, rheumatoid arthritis; SD, standard deviation; RF, rheumatoid factor; anti-CCP Ab, anti-cyclic citrullinated peptide antibody; CRP, C-reactive protein; ESR, erythrocyte sedimentation rate, BMI: body mass index. Anti-CCP Ab ≥ 20 IU/mL^28^, RF ≥ 20 IU/mL^29^, CRP > 0.5 mg/dL, ESR > 15 mm/h in males and ESR > 20 mm/h in females^30^ were considered ‘positive’ or above the normal range. Statistical significance was set at p-value < 0.05. *p < 0.05, **p < 0.01, ***p < 0.001, significant values are mentioned in bold.

### Distribution of obesity and diabetes mellitus in OA and RA

The body mass index (BMI) values and the prevalence of diabetes mellitus (DM) were compared between OA and RA patients. DM cases were only considered if diagnosed by a physician and reported by the patients during history taking on their systemic diseases. There was no significant difference in BMI values between OA and RA patients (24.58 ± 3.33 kg/m^2^ vs. 23.81 ± 3.84 kg/m^2^, *p* = 0.321). As per criteria established in previous studies regarding BMI, patients were categorized as normal with a BMI < 25 kg/m^2^, overweight for BMI ≥ 25 kg/m^2^ to < 30 kg/m^2^, and obesity for BMI ≥ 30 kg/m^2^^36,37^. Overweight was notably higher in OA patients compared to RA patients (80.6% vs 61.9%, *p* = 0.041), while there was no significant difference observed in obesity between the two groups (3.2% vs. 4.1%, *p* = 0.137). The prevalence of DM was significantly higher in OA patients compared to RA patients (29.0% vs 10.3%, *p* = 0.015) (Table 2).

### Comparison of snRA and spRA subtypes

When comparing the demographics and oral health of patients with snRA and spRA, the age of patients with spRA (58.04 ± 11.63 years) was significantly higher than that of those with snRA (52.33 ± 14.37 years) (Table 3). There was no difference in sex distribution between the two RA subgroups. More number of women had both snRA (83.3%) and spRA (87.3%) than men. All patients with snRA and 91.1% patients with spRA had periodontal diseases. Periodontitis was observed more frequently than gingivitis in both snRA (22.2% vs. 77.8%) and spRA (19.0% vs. 72.2%) groups (Fig. 2B). There was no significant difference in the distribution of periodontal diseases between the two RA subtypes (*p* > 0.05). In the context of VSC levels, H_2_S and VSC sum did not differ between patients with snRA and spRA. However, CH_3_SH level was significantly higher in patients with snRA (39.39 ± 62.05 ppb) than in those with spRA (19.68 ± 29.12 ppb) (*p* = 0.045). The incidence of halitosis was significantly higher in the snRA group (55.6%) than in the spRA group (30.4%) (*p* = 0.042) (Fig. 2B and Table 3). The salivary flow rate of patients with spRA (0.16 ± 0.29 mL/min) was significantly lower than those with snRA (0.17 ± 0.42 mL/min) (*p* = 0.022). Xerostomia was highly prevalent in patients with snRA (72.2%) and spRA (87.3%); however, the difference between the two subgroups was not significant (*p* = 0.111).

**Table 3:** Comparison of demographics and oral health of patients with snRA and spRA.

	snRA (n = 18)	spRA (n = 79)	*p*-value
	Mean ± SD or n (%)	Mean ± SD or n (%)	
Demographics			
Age (years)^a^	52.33 ± 14.37	58.04 ± 11.63	0.022*
Sex^b^			
Male	3 (16.7%)	10 (12.7%)	0.703
Female	15 (83.3%)	69 (87.3%)	
Non-periodontal disease^b^	0 (0%)	7 (8.9%)	0.342
Periodontal diseases^b^	18 (100%)	72 (91.1%)	0.420
(1) Gingivitis	4 (22.2%)	15 (19.0%)	
(2) Periodontitis	14 (77.8%)	57 (72.2%)	
VSC level (ppb)			
(1) H_2_S^a^	51.61 ± 55.50	44.69 ± 69.43	0.694
(2) CH_3_SH^a^	39.39 ± 62.05	19.68 ± 29.12	0.045*
(3) VSC sum^a^	97.39 ± 100.56	64.38 ± 93.93	0.187
Halitosis^b^	10 (55.6%)	20 (30.4%)	0.042*
Salivary flow rate (mL/min)^a^	0.17 ± 0.42	0.16 ± 0.29	0.022*
Xerostomia^b^	13 (72.2%)	69 (87.3%)	0.111

^a^Results were obtained using t-test. ^b^Results were obtained using two-sided Chi-square analysis. snRA, seronegative rheumatoid arthritis; spRA, seropositive rheumatoid; SD, standard deviation. Statistical significance was set at *p*-value < 0.05. *p < 0.05, **p < 0.01, ***p < 0.001, significant values are mentioned in bold.

### Comparison of hematological parameters between snRA and spRA

When examining snRA and spRA subtypes, the average levels of anti-CCP Ab, RF, and CRP were significantly higher in spRA than in snRA (all *p < *0.05) (Table 4). Anti-CCP Ab was significantly higher in spRA (531.60 ± 637.14 IU/mL) than snRA (13.83 ± 8.00 IU/mL) (*p < *0.001). Similarly, RF was significantly higher in spRA (134.48 ± 194.37 IU/mL) than in snRA (10.17 ± 1.72 IU/mL) (*p* = 0.003). Furthermore, CRP was also significantly higher in spRA (0.96 ± 0.19 mg/dL) than in snRA (0.28 ± 0.46 mg/dL) (*p < *0.001). Of the 79 patients with spRA, 65 (82.3%) were positive for both anti-CCP Ab and RF, and 17.7% were positive for only one. Most patients with spRA were positive for anti-CCP Ab (98.7%) and RF (82.3%); whereas none of the patients with snRA were positive for both anti-CCP Abs or RF. Cases with CRP and ESR positivity were found at a higher rate in the spRA group than in the snRA group; however, the difference was not statistically significant.

**Table 4:** Hematological characteristics of patients with snRA and spRA.

	snRA (n = 18) Mean ± SD or n (%)	spRA (n = 79) Mean ± SD or n (%)	*p*-value
Anti-CCP Ab (IU/mL)^a^	13.83 ± 8.00	531.60 ± 637.14	< 0.001***
Anti-CCP Ab positive^b^	0 (0.0%)	78 (98.7%)	< 0.001***
RF (IU/mL)^a^	10.17 ± 1.72	134.48 ± 194.37	0.003**
RF positive^b^	0 (0.0%)	65 (82.3%)	< 0.001***
CRP (mg/dL)^a^	0.28 ± 0.46	0.96 ± 0.19	< 0.001***
CRP positive^b^	3 (16.7%)	5 (6.3%)	0.192
ESR (mm/hr)^a^	24.06 ± 17.22	23.11 ± 15.44	0.496
ESR positive^b^	10 (55.6%)	45 (57.0%)	0.530

^a^Results were obtained using t-test. ^b^Results were obtained using two-sided Chi-square analysis (two-sided). snRA, seronegative rheumatoid arthritis; spRA, seropositive rheumatoid arthritis; SD, standard deviation; anti-CCP Ab, anti-cyclic citrullinated peptide antibody; RF, rheumatoid factor; CRP, C-reactive protein; ESR, erythrocyte sedimentation rate. Anti-CCP Ab ≥ 20 IU/mL^28^, RF ≥ 20 IU/mL^29^, CRP > 0.5 mg/dL, ESR > 15 mm/h in males and ESR > 20 mm/h in females^30^ were considered ‘positive’ or above the normal range. Statistical significance was set at p-value < 0.05. *p < 0.05, **p < 0.01, ***p < 0.001. Significant values are mentioned in bold.

### ROC analysis in the prediction of OA and RA

The AUC values for diagnosing or predicting OA and RA based on ROC analysis under each condition are presented in Table 5. First, we selected factors that could significantly predict one group compared with other groups based on conventional statistical results that examined differences in means and distributions.

**Table 5:** Receiver operating curve analysis in diagnosing OA and RA.

	AUC	95% CI lower	95% CI upper	*p*-value
OA diagnosis compared to healthy controls				
Age	0.516	0.373	0.659	0.825
Female sex	0.663	0.529	0.797	0.025*
Periodontal diseases	1.000	1.000	1.000	< 0.001***
Halitosis	0.746	0.621	0.871	0.0007***
Xerostomia	0.516	0.373	0.659	0.825
RA diagnosis compared to healthy controls				
Age	0.923	0.877	0.968	< 0.001***
Female sex	0.660	0.544	0.776	0.006**
Periodontal diseases	0.964	0.933	0.995	< 0.001***
Halitosis	0.615	0.510	0.719	0.049*
Xerostomia	0.923	0.877	0.968	< 0.001***
OA diagnosis compared to RA				
Age	0.631	0.518	0.745	0.028*
Halitosis	0.631	0.518	0.745	0.028*
RA diagnosis compared to OA				
Xerostomia	0.900	0.834	0.966	< 0.001***
Anti-CCP Ab	0.808	0.701	0.915	< 0.001***
RF	0.746	0.639	0.854	< 0.001***
snRA diagnosis compared to spRA				
Xerostomia	0.575	0.420	0.730	0.324
Halitosis	0.370	0.222	0.517	0.086
Anti-CCP Ab	1.000	1.000	1.000	< 0.001***
RF	0.910	0.854	0.967	< 0.001***

Results were obtained using receiver operating characteristic (ROC) curve analysis. AUC, area under the curve; CI, confidence interval; OA, osteoarthritis; RA, rheumatoid arthritis; snRA, seronegative rheumatoid arthritis; spRA, seropositive rheumatoid arthritis; anti-CCP Ab, anti-cyclic citrullinated peptide antibody; RF, rheumatoid factor. Statistical significance was set at p-value < 0.05. *p < 0.05, **p < 0.01, ***p < 0.001. Significant values are marked in bold.

In contrast to healthy controls, periodontal disease was a very strong predictor in diagnosing OA, with an AUC value of 1.00 (*p < *0.001). Additionally, halitosis (AUC = 0.746, 95% CI 0.621–0.871, *p < *0.001) and female sex (AUC = 0.663, 95% CI 0.529–0.797, *p < *0.05) were also found to be significant predictors of OA.

In contrast to healthy controls, the strongest predictors of RA were periodontal diseases (AUC = 0.964), followed by xerostomia (AUC = 0.923), age (AUC = 0.923), female sex (AUC = 0.660), and halitosis (AUC = 0.615) (all *p < *0.05). Periodontal diseases, xerostomia, and age showed outstanding performances in predicting RA (AUC > 0.9).

In diagnosing or predicting OA compared to RA, the significant factors were age (AUC = 0.631) and halitosis (AUC = 0.631), and their predictive power was acceptable (0.7 > AUC > 0.6).

In diagnosing RA compared to OA, the significant predictors were xerostomia (AUC = 0.900), anti-CCP Ab (AUC = 0.808), and RF (AUC = 0.746) (all *p < *0.05). Xerostomia and anti-CCP Ab showed excellent prediction performance in predicting RA. To predict RA compared to OA, we conducted additional multiple logistic regression analysis (Table 6). After adjusting for age, sex, BMI-based overweight, and diabetes mellitus, adjusted p-values were derived, considering the notable clinical differences observed in these variables between OA and RA patients. As a result, significant predictors for RA included xerostomia (OR: 8124.879, 95% CI 10.366–6,368,261.974, *p*-value = 0.008) and Anti-CCP Ab (OR: 671.331, 95% CI 2.176–207,074.023, *p* = 0.026), while interestingly, RF was not a significant predictor.

**Table 6:** Results of multiple logistic regression analysis of RA compared to OA.

Predictor variables	Outcome variable: the presence of RA				
	OR	95% CI_upper	95% CI_lower	Regression coefficient	Adjusted p-value
Periodontal diseases	13.345	0.100	1777.983	2.591	0.299
Halitosis	19.266	0.574	646.371	0.335	0.099
Xerostomia	8124.879	10.366	6,368,261.974	9.003	0.008**
Anti-CCP Ab	671.331	2.176	207,074.023	6.509	0.026*
RF	1.398	0.086	22.794	0.335	0.814

The results were obtained using multiple logistic regression analysis (R^2^: 0.869, adjusted R^2^: 0.847). After adjusting for age, sex, BMI-based overweight, and diabetes mellitus, adjusted p-values were obtained, considering the notable clinical differences on these variables observed between osteoarthritis and rheumatoid arthritis patients. RA, rheumatoid arthritis; OA, osteoarthritis; OR: odds ratio, CI confidence interval, anti-CCP Ab, anti-cyclic citrullinated peptide antibody; RF, rheumatoid factor. Statistical significance was set at *p*-value < 0.05. *p < 0.05, **p < 0.01. Significant values are marked in bold.

**Figure 4:**
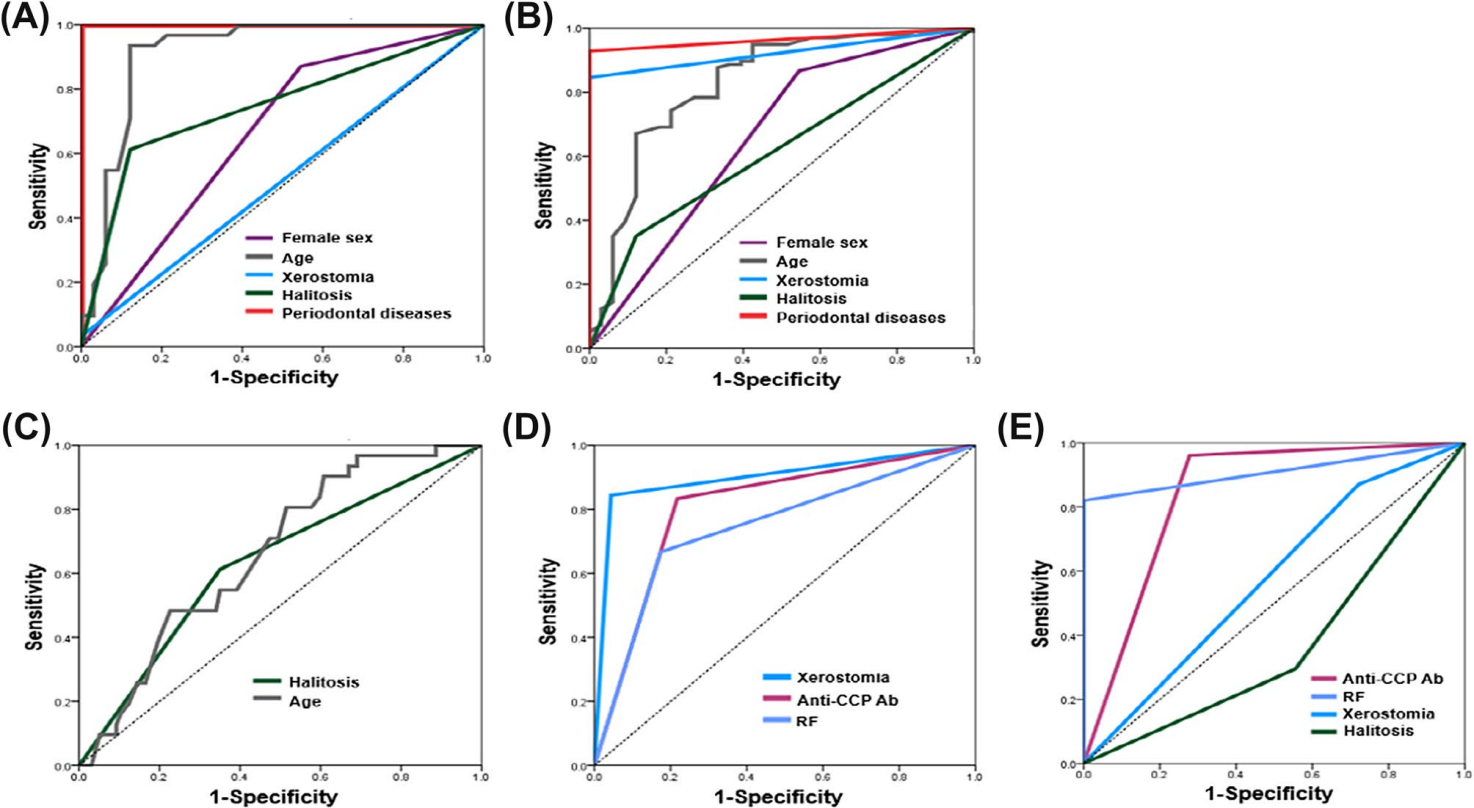
ROC curves of significant factors for OA and RA. (**A**) OA diagnosis compared to healthy controls, (**B**) RA diagnosis compared to healthy controls, (**C**) OA diagnosis compared to RA, (**D**) RA diagnosis compared to OA, (**E**) snRA compared to spRA.

When diagnosing spRA compared to snRA, anti-CCP Ab (AUC = 1.000, *p < *0.001) and RF (AUC = 0.910, 95%CI 0.854–0.967, *p < *0.001) demonstrated outstanding prediction performances for spRA compared to snRA. Factors related to oral health, such as halitosis and xerostomia, were not significant predictors of spRA compared to snRA. Only serological factors, including anti-CCP and RF, were found to be significant predictors (Fig. 4). No significant factor that distinguishes OA from snRA was identified.

### Relationship between oral health parameters and hematological factors

Cramer’s V analysis was performed to determine the relationship between the nominal variables. Cramer’s V (Cramer's coefficient) refers to the strength of association between two nominal variables and is distributed from 0 to 1, and the closer it is to 1, the higher is the correlation^38^. The presence of xerostomia was significantly associated with anti-CCP Ab (Cramer’s V = 0.379, *p < *0.001) and RF (Cramer’s V = 0.435, *p < *0.001) positivity. RF was not only significantly related to xerostomia, but also to halitosis (Cramer’s V = 0.184, *p* = 0.042) and periodontitis (Cramer’s V = 0.217, *p* = 0.016). Among the hematological indicators, anti-CCP Ab and RF demonstrated a strong significant correlation with each other (Cramer’s V = 0.542, *p < *0.001). CRP and ESR showed a weak relationship with each other (Cramer’s V = 0.193, *p* = 0.029) (Table 7).

**Table 7:** Relationship between oral health parameters and serological factors.

		Halitosis (n = 161)	Periodontal diseases (n = 161)	Anti-CCP Ab (n = 128)	RF (n = 128)	CRP (n = 128)	ESR (n = 128)
Xerostomia	Cramer’s V	0.114	0.421**	0.379**	0.435**	0.074	0.074
	*p*-value	0.150	0.000	0.000	0.000	0.403	0.406
Halitosis	Cramer’s V		0.215**	0.109	0.184*	0.107	0.045
	*p*-value		0.006	0.237	0.042	0.229	0.610
Periodontal diseases	Cramer’s V			0.146	0.217*	0.066	-0.016
	*p*-value			0.114	0.016	0.458	0.861
Anti-CCP Ab	Cramer’s V				0.542**	0.101	0.080
	*p*-value				0.000	0.277	0.385
RF	Cramer’s V					0.066	0.112
	*p*-value					0.468	0.216
CRP	Cramer’s V						0.193*
	*p*-value						0.029

Results were obtained using Cramer’s V analysis. Anti-CCP Ab, anti-cyclic citrullinated peptide antibody; RF, rheumatoid factor; CRP, C-reactive protein; ESR, erythrocyte sedimentation rate. Statistical significance was set at p-value < 0.05. *p < 0.05, **p < 0.01, ***p < 0.001. Significant values are marked in bold.

## Discussion

To the best of our knowledge, this study is the first that sought to discover new biomarkers for OA and RA by conducting a comparative analysis among patients with these disease entities and healthy controls. All participants were examined for oral health, including periodontal diseases, halitosis, and xerostomia. For halitosis, VSC was directly measured, and for xerostomia, unstimulated salivary flow rate was measured. Most patients with OA (100%) and RA (92.8%) had periodontal diseases, and the proportion of patients with periodontitis, a more advanced form than gingivitis, was higher in patients with OA (12.9% vs. 87.1%) and RA (19.6% vs. 19.6%). 73.2%). Halitosis was a significant predictor of OA compared with healthy controls and patients with RA. Additionally, patients with RA had a significantly lower salivary flow rate and an extremely higher rate of xerostomia than healthy controls and patients with OA, and xerostomia was a significant predictor of RA. Anti-CCP and RF, which traditionally distinguish patients with snRA from those with spRA, were also found to be useful biomarkers in this study. There was a significant relationship between occurrence of oral diseases and hematological abnormalities.

OA is the most common type of arthritis and causes chronic disabilities. OA most commonly affects large weight-bearing joints, such as the hands, feet, knees, hips, and spine. The prevalence of arthritis and disability rate among people aged above 75 years has been reported to be 80% and 53%, respectively^39^. The accuracy of OA diagnosis varies depending on the diagnostic tool used and disease severity. When diagnosing OA using plain radiography, the sensitivity and specificity reportedly ranges between 3.0 and 95.0% and 60.0–98.0%^40,41^. The Kellgren-Lawrence system has a sensitivity of 95.0% when diagnosing severe osteoarthritis, which is higher than the 83.0% sensitivity for joint space narrowing^41^. However, joint space narrowing depends on joint curvature and X-ray beam position^42^, and the results must be interpreted with caution. In the context of hematological factors, increased ESR and CRP levels have been reported to be associated with increased symptom severity in patients with OA^43,44^. However, there have been reports that CRP is related to symptom severity in patients with OA, but ESR reportedly has no significance^45^. In this study, ESR and CRP levels showed weak correlations with each other. However, ESR and CRP levels were not investigated in healthy controls; therefore, OA could not be predicted compared to healthy controls using these parameters.

RA is a chronic, multisystemic autoimmune disease of unknown origin. RA, characterized by chronic joint inflammation, primarily affects the lining of synovial joints, which may later develop into joint destruction and functional limitations. In this study, ESR and CRP levels were found to be significantly higher in patients with RA than in those with OA. Elevation in ESR and CRP levels are the most commonly considered acute-phase reactants^46^. However, several studies have indicated similar alterations in ESR and CRP levels in various other diseases including systemic lupus erythematosus and other systemic infections^47,48^. Serologic test results for RF and anti-CCP Ab along with various clinical signs and symptoms based on the patient’s reports and rheumatologist's professional judgment are required for confirmatory testing of RA. In 2010, the ACR and EULAR added anti-CCP Ab, also known as ACPA, a biomarker predicting aggressive RA, to the existing biomarker, RF, and revised the classification criteria to emphasize the characteristics of early RA^49^. Recently, the detection of autoantibodies such as RF and anti-CCP Ab has provided an important basis for early diagnosis and assessment of disease activity^50,51^. In this study, the significant predictors of RA compared to OA were xerostomia (AUC = 0.900), anti-CCP Ab (AUC = 0.808), and RF (AUC = 0.746). Anti-CCP Ab and RF showed excellent and acceptable performance in predicting RA.

RF and anti-CCP Ab are the most commonly used serum markers for diagnosing RA. Approximately 70% of patients with RA test positive for RF at the onset of RA. RF is present in only 70%–80% of patients with RA and can be nonspecific^52,53^. Anti-CCP Abs can be detected in the serum of 60–80% patients with RA^54^. However, the sensitivity of anti-CCP Ab and RF has been reported to be 61.8% and 64.4%, respectively. On the other hand, specificity of anti-CCP Ab and RF reportedly is 91.95% and 76.51%, respectively^55^. This suggests that anti-CCP Ab has an advantage over RF in terms of specificity. The specificity of RF in RA is lower than that of anti-CCP Abs, as positivity for RF can occur in several rheumatic or immune diseases, including Sjögren's syndrome, systemic lupus erythematosus, and primary cryoglobulinemia, as well as in viral infections or tumors^56^. Moreover, anti-CCP Ab levels have been reported to be closely associated with progression of early RA^57^. In the present study, RF and anti-CCP Ab had a strong and significant correlation with each other. Further investigation is needed to determine how RF and anti-CCP Abs play a role in the clinical characteristics of patients with RA, as well as RA cases in which RF and anti-CCP Abs were simultaneously detected, and RA cases in which neither was detected.

In this study, of a total of 97 patients with RA, 18 (18.6%) had snRA and 79 (81.4%) had spRA, with an snRA:spRA ratio of 1:4.39. As previously mentioned, 20–30% of patients with RA do not have RF and/or anti-CCP Abs. Seronegative RF may indicate the presence of low levels of antibodies that do not warrant seropositivity typically observed in RA^58^. Diagnosis of RA depends on a set of clinical signs and symptoms according to the 2010 ACR-EULAR classification^49^. According to this scoring definition, if a patient obtains a score of ≥ 6/10, they are diagnosed with definite RA. However, in this scoring system, the maximum scores assigned to RF and anti-CCP Ab is 3. This means that the diagnosis may be missed or delayed in patients with seronegative RF, and lead to disease progression. This destructive process causes patients with snRA to suffer continuously. However, diagnosing seronegative RA remains challenging. Ultimately, snRA is a clinical diagnosis that may be difficult to definitively distinguish from spRA^59^. The predictive performance of anti-CCP Ab and RF was outstanding when diagnosing spRA compared to snRA; however, oral health-related factors did not distinguish between these two RA subsets. According to Disale et al., severe periodontitis occurred more frequently in patients with spRA (69.0%) than in those with snRA (16.6%)^60^. However, in the present study, there was no significant difference in the distribution of periodontal diseases between the snRA and spRA groups.

The strength of this study is that it investigated whether oral health-related factors, in addition to hematological factors, were significant predictors of the diagnosis of OA or RA. In contrast to healthy controls, periodontal disease was found to be a very strong predictor of OA. Moreover, halitosis and female sex were also significant predictors of OA. Furthermore, periodontal diseases, xerostomia, and age showed outstanding performance in predicting RA. A bidirectional relationship has been reported between OA and periodontal diseases^61^. The mechanism by which these two diseases are connected is not clear; however, it can be explained as follows. Periodontitis-induced oral inflammation influences the development and progression of OA via several virulence factors. Periodontitis is initiated by the formation of dysbiotic biofilms that trigger an inflammatory response in the host via expression of inflammatory cytokines^62^. Although the oral and joint spaces are far apart, they are connected through systemic inflammatory responses. Periodontal pathogens such as *Porphyromonas gingivalis* (*P.gingivalis*) can spread to the joints through the bloodstream, and the same bacterial DNA has been found in the periodontal tissue and synovial fluid of patients with OA^63^. Therefore, periodontal bacteria may contribute to joint inflammation and damage. Moreover, both periodontitis and OA are mediated by proinflammatory cytokines such as interleukin (IL)-1β and tumor necrosis factor (TNF)-α^64^. Levels of proinflammatory cytokines such as IL-6 and TNF-α are elevated in the synovial fluid and cartilage of patients with OA. These proinflammatory cytokines upregulate the inflammatory response and inhibit proteoglycan and type II collagen synthesis in chondrocytes^65^. Similar to the mechanism of periodontitis in OA, in RA, the upregulation of systemic inflammatory response due to periodontal diseases or the adverse effects of *P.gingivalis* may be the link between the two diseases^66^.

Research on salivary flow rate, xerostomia, and hyposalivation in patients with RA has been surprisingly limited. In this study, 87.3% of patients with RA had xerostomia. In RA, the salivary glands are profoundly affected, and the destruction of the salivary glands and ductal system can reduce the patient's quality of life^67^. Our results showed that xerostomia, along with aging, was a major predictor of RA; however, multicenter studies with larger number of participants are needed to confirm our findings. In the context of the relationship between xerostomia and halitosis, only the general perception, that is, the opinion that xerostomia can cause halitosis, has been introduced^68^. In the present study, xerostomia and halitosis were not found to be significantly correlated. Occurrence of halitosis increases with the increase in VSC level, and a specific VSC cutoff value is used as a diagnostic criterion for halitosis^27,69^. Representative VSCs include H_2_S and CH_3_SH, which account for more than 90% of all VSCs^70^. As there has been little research on oral health, such as xerostomia and halitosis, in patients with OA and RA by measuring salivary flow rate and VSC levels, additional research is warranted to identify and clarify the predictors of OA and RA along with the factors that may aid in diagnosis.

This study had a few limitations that need consideration. First, we examined OA and RA by dichotomously categorizing them into presence or absence, and no analysis was performed based on either disease stage or severity. In addition, several pro-inflammatory cytokines, autoimmune-related antibodies, genetic factors, or environmental factors may be related to OA and RA; however, not all these factors were investigated. Furthermore, there was a difference in the number of patients constituting the groups when comparing OA and RA. The number of participants included in the snRA and spRA groups for comparison was also not similar, and the number of patients with snRA was less than 20. As per the sample size calculation, a minimum of 30 patients per group was deemed necessary. This discrepancy raises concerns regarding adequate statistical power and the potential for false positives. Diagnosing snRA is challenging, and it was difficult to obtain consent from patients who complained of physical fatigue or weakness to complete the research protocol. As this study is conducted within a single institution rather than a national cohort, further analysis is warranted to encompass representatives of typical RA and OA patients in Korea. Despite these limitations, our study is the first to objectively compare the oral health and diseases of patients with OA and RA and healthy controls. Our study also compared the increased occurrence of periodontal diseases in patients with OA and RA through the judgment of experts in each field.

In conclusion, this study explored hematological characteristics and various oral health-related factors among OA and RA patients, demonstrating their utility in predicting and diagnosing these conditions. That is, oral health-related factors were assessed for their potential predictive value in distinguishing OA and RA from healthy controls. Notably, halitosis and xerostomia emerged as significant predictors for OA and RA, respectively. Therefore, clinicians and researchers should scrutinize the oral status of OA and RA patients, considering both oral health-related factors and serological markers as crucial predictors. Despite attempts to differentiate between snRA and spRA using oral health-related factors, significant distinction primarily relied on traditional serological markers such as anti-CCP Ab and RF. Diagnosis of snRA in clinical settings relies on classification criteria by experienced rheumatologists, lacking objective biological indicators. To bolster these findings, further validation through a national or multi-center cohort study involving a larger participant cohort is essential, along with international verification using consistent research methodologies across various countries.

## Data Availability

The datasets used and/or analyzed in the current study are available from the corresponding author upon reasonable request.
